# GmbZIP1 negatively regulates ABA-induced inhibition of nodulation by targeting *GmENOD40–1* in soybean

**DOI:** 10.1186/s12870-020-02810-9

**Published:** 2021-01-09

**Authors:** Shimin Xu, Shanshan Song, Xiaoxu Dong, Xinyue Wang, Jun Wu, Ziyin Ren, Xuesong Wu, Jingjing Lu, Huifang Yuan, Xinying Wu, Xia Li, Zhijuan Wang

**Affiliations:** National Key Laboratory of Crop Genetic Improvement, College of Plant Science and Technology, Huazhong Agricultural University, Wuhan, Hubei 430070 P.R. China

**Keywords:** ABA, Nodulation, GmbZIP1, *GmENOD40–1*, Soybean

## Abstract

**Background:**

Abscisic acid (ABA) plays an important role in plant growth and adaptation through the ABA signaling pathway. The ABA-responsive element binding (AREB/ABF) family transcriptional factors are central regulators that integrate ABA signaling with various signaling pathways. It has long been known that ABA inhibits rhizobial infection and nodule formation in legumes, but the underlying molecular mechanisms remain elusive.

**Results:**

Here, we show that nodulation is very sensitive to ABA and exogenous ABA dramatically inhibits rhizobial infection and nodule formation in soybean. In addition, we proved that GmbZIP1, an AREB/ABF transcription factor, is a major regulator in both nodulation and plant response to ABA in soybean. *GmbZIP1* was specifically expressed during nodule formation and development. Overexpression of *GmbZIP1* resulted in reduced rhizobial infection and decreased nodule number. Furthermore, *GmbZIP1* is responsive to ABA, and ectopic overexpression of *GmbZIP1* increased sensitivity of Arabidopsis plants to ABA during seed germination and postgerminative growth, and conferred enhanced drought tolerance of plants. Remarkably, we found that GmbZIP1 directly binds to the promoter of *GmENOD40–1*, a marker gene for nodule formation, to repress its expression.

**Conclusion:**

Our results identified GmbZIP1 as a node regulator that integrates ABA signaling with nodulation signaling to negatively regulate nodule formation.

**Supplementary Information:**

The online version contains supplementary material available at 10.1186/s12870-020-02810-9.

## Background

Root nodules are specialized root organ in leguminous plants developed under low nitrate conditions, where legumes and nitrogen-fixing bacteria (rhizobia) form a symbiotic relationship. In symbiotic nodules, legume plants provide bacteria with carbohydrates and energy for its growth and survival, conversely bacteria supply the plants with NH3 converted from the atmospheric N_2_ to meet the high nitrogen demand of legume plants [1–3].

The process of nodulation is initiated by a reciprocal and specific crosstalk between legumes and rhizobia. Under low nitrogen conditions, legume roots secrete flavonoid molecules into rhizosphere, which induce compatible rhizobia to synthesize and release signaling molecules called Nod factors (NFs) [4–6]. NFs are perceived by Lysine motif receptor-like kinases (LysM) receptors (NFRs) located at plasma membrane of the epidermal cells of roots and root hairs. These NF receptors include NF Perception (NFP) in *M. truncatula*, NF Receptors 1 and 5 (NFR1/5) in *L. japonicus*, SYM10/SYM2 in *P. sativum* (pea) and NFR1α/β and NFR5α/β in *G. max* (soybean) [7–11]. The perception of NFs by these LysM receptors triggers a signaling cascade (the nodulation signaling) and a variety of cellular events to activate rhizobial infection and nodule formation. The key events following the NF perception are calcium spiking in the cytoplasm of infected root hair cells and transducing of the calcium signal to the nucleus by Does Not Make Infections 1 and 2 (DMI1 and DMI2) and Nuclear-localized cyclic nucleotide-gated channels (CNGC15) [12–14]. Calcium oscillations in the nucleus is decoded by Calcium and Calmodulin-dependent Kinase (CCaMK in Lotus and DMI3 in Medicago) that form complex with CYCLOPS to activate a set of transcriptional factors [15, 16]. These transcriptional factors include two GRAS domain transcriptional regulators [17–21], including Nodulation Signaling Pathway1 (NSP1) and NSP2, which can form a complex to directly activate expression of *Nodule Inception* (*NIN*), *Ethylene Response Factor Required for Nodulation1* (*ERN1*) and *Early Nodulin 11* (*ENOD11*) [22, 23].

Phytohormones play a central role in symbiotic nodulation. Among them, cytokinin (CK) and auxin positively regulate symbiotic nodulation [24–29], while other hormones, such as jasmonate (JA), salicylic acid (SA), gibberellic acid, ethylene and ABA negatively regulate both rhizobial infection and nodule organogenesis [30]. Although ABA is well-known for its anti-stress effects in plants [31], researchers have also identified a negative role of ABA in nodulation of legumes. Exogenous application of ABA strongly reduced nodule number, while the treatment of Abamine, a specific ABA biosynthesis inhibitor increased nodule number [32–36]. ABA inhibited NF-induced calcium spiking in *M. trunctula*, suggesting that ABA affects nodulation at very early stage [35]. In *L. japonicus,* ABA treatment dramatically decreased the number of curled root hairs and the infection threads [33]. Consistent with these results, the induction of *nodulin* genes by NFs or rhizobia, such as *Rhizobium-induced Peroxidase* (*RIP*) and *ENOD11*, was abolished by ABA treatment [35]. Further genetic evidence that blocking the ABA signaling by overexpression of the Arabidopsis dominant negative allele *abi1–1* increased *nodulin* genes expression and subsequent nodule number demonstrates an inhibitory role of ABA in nodulation [35]. It appears that ABA suppressed the cytokinin-activated expression of a nodulation gene *ENOD40* that is involved in nodule formation in root cortex [35], although the detailed mechanisms by which ABA inhibits nodulation and cross-talks to cytokinin are still to be elucidated.

ABA exerts its function through the canonical ABA signaling, which is a dynamic process that is initiated by the binding of ABA to the ABA receptors (the PYR1/PYLs/RCAR protein family), and involved the rapid transmission of the signal by group A protein type 2C phosphatases (PP2Cs) and sucrose nonfermenting-1-related protein kinase class 2 (SnRK2s) to the nucleus followed by ultimate activation of transcription factors resulting in altered expression of target genes [37–42]. ABA-responsive element (ABRE) binding protein/ABRE binding factors (AREB/ABF) transcription factors are responsible for the expression of most of the target genes, which are also known as ABRE/ABF regulon. In plants, these AREB/ABF transcription factors are basic region/leucine zipper (bZIP) class family proteins and can bind to the ABRE *cis* element in the promoters of ABA-responsive genes [43–45]. As a result, ABA can help to regulate plant response to abiotic stress [43–45]. Thus, the AREB/ABF transcription factors play a crucial role in controlling the activity of genes that regulate various aspects of plant response.

The inhibitory effect of ABA on soybean nodulation has been reported [32, 46–48]. It has been shown that ABA treatment markedly reduced production of isoflavonoid compounds, such as daizdzein, genistein and coumestrol [32], thereby leading reduced rhizobial infection. However, no significant progress has been made in how ABA affects soybean nodulation since then. Based on the current evidence, soybean has the canonical ABA signaling, which consists of PYR1/PYLs receptors for ABA, PP2Cs, SnRKs and AREB/ABF transcription factors. For GmbZIPs, there are 160 GmbZIP genes identified in soybean genome, and few of them (e.g. *GmbZIP1* and *GmbZIP2*) has been functionally analyzed for their role in ABA response and stress tolerance [49, 50]. However, the role of the AREB/ABF transcription factors and the molecular mechanism by which ABA interplays with nodulation signaling remain completely unknown. In this study, we analyzed the effects of ABA on soybean growth and nodulation. We identified GmbZIP1, a soybean homolog of Arabidopsis AREB1, as a positive regulator of ABA response, but a negative regulator of soybean nodulation. *GmbZIP1* was induced by ABA and ectopic expression of *GmbZIP1* in Arabidopsis enhanced ABA sensitivity and plant tolerance to drought. Importantly, *GmbZIP1* was highly expressed in nodule, and overexpression of *GmbZIP1* resulted in reduced nodulation. Furthermore, we found that *GmbZIP1* directly bound to and repressed the expression of *GmENOD40–1*. These findings reveal for the first time the interplay between ABA and nodulation signal transduction pathway that fine tunes rhizobial infection and nodule formation in soybean.

## Results

### ABA inhibits nodulation in soybean

**Fig. 1:**
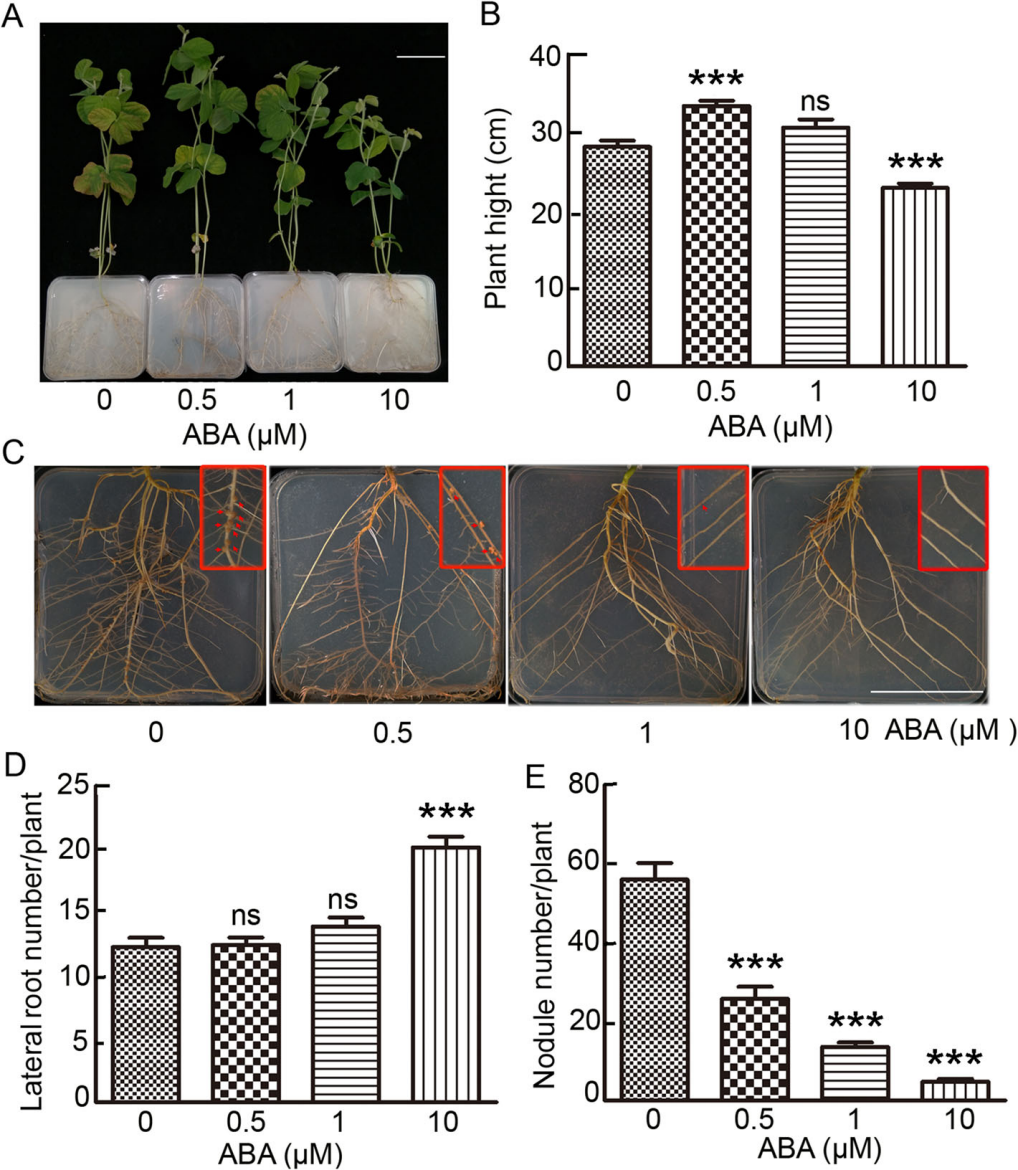
Exogenous ABA treatment reduces nodule number in soybean. **a** The phenotype of the aerial part of soybean treated with 0, 0.5, 1 or 10 μΜ ABA; Bar=7 cm; **b** Comparison of plant height of soybean with different concentration of ABA; **c** The phenotype of root and nodule of soybean with different concentration of ABA; **d** Comparison of primary lateral roots number of soybean treated with different concentration of ABA; **e** Comparison of root nodule number of soybean treated with different concentration of ABA. Bar = 7 cm. Values are means ± SD. ***, *P* < 0.001 and ns, *P* > 0.05.

To assess the role of ABA during symbiotic nodulation in soybean, we first analyzed ABA effects on plant growth and nodule formation. Six day-old soybean seedlings germinated on agar media were transferred onto Jensen’s plates [51] supplemented with 0, 0.5, 1 or 10 μM ABA and inoculated the plants with *B. diazoefficiens* USDA110. Eighteen days after ABA treatment and rhizobial inoculation, plant height, lateral root number and the number of root nodules were evaluated. As shown in Fig. 1a and b, shoot growth of the soybean seedlings was sensitive to ABA. Shoot growth was promoted by low concentration (0.5 μM) of ABA but was inhibited by high concentration (10 μM) of ABA. In comparison, lateral root formation of soybean seedlings showed different pattern in the conditions of ABA treatment (Fig. 1c and d). Lateral root formation of the plants treated with low concentrations (0.5 and 1 μM) of ABA was comparable to that of the control plants, however, lateral roots were significantly increased when treated with high concentration (10 μM) of ABA (Fig. 1c and d). In sharp contrast, nodulation was quite sensitive to ABA in soybean and nodule number strongly diminished with increased concentrations of ABA (Fig. 1c and e). The nodule number per plant under 0.5 μM ABA treatment was reduced to half of the control plants, and the nodule number was nearly completely abolished in the presence of 10 μM ABA. These results indicate that root nodule is the most sensitive organ of soybean to ABA during growth and development, and ABA plays a negative regulatory role in soybean nodulation.

### ABA inhibits Rhizobial infection and NF signaling

**Fig. 2:**
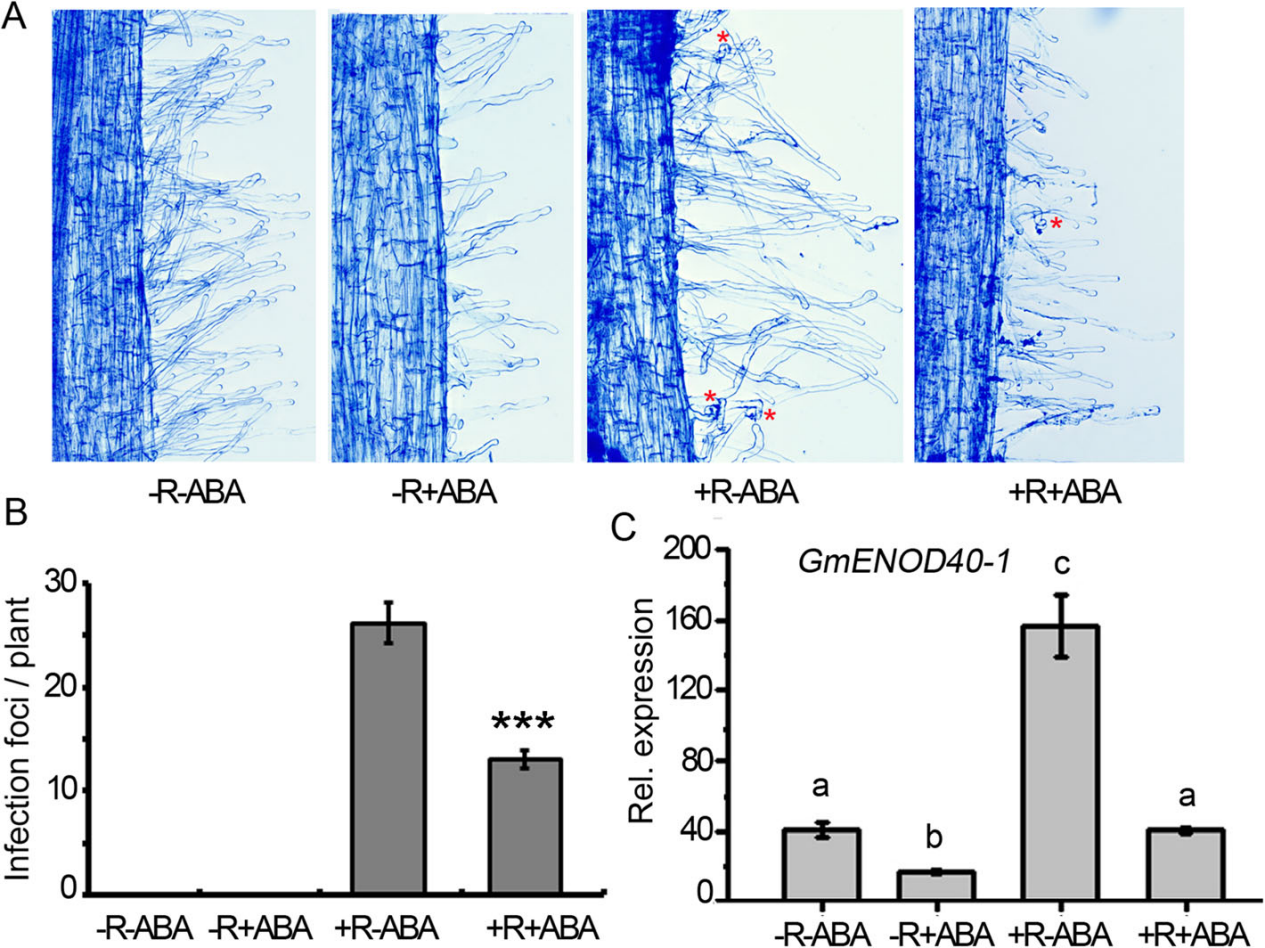
ABA inhibits reciprocal recognition of soybean and rhizobia. **a** Curled root hair of roots with ABA and rhizobia treatment. The asterisk marks the curled root hair at 7 day after inoculation with rhizobia; **b** The infection foci number per plant (*n*=6). Values are means ± SD. *** *P*< 0.001; **c** Expression of *GmENOD40–1* in the treatment of ABA and/ or rhizobia. The *GmENOD40–1* was detected in roots at 3 DAI with USDA110. -R-ABA: treatment without rhizobia or ABA; −R+ABA: treatment without rhizobia but with ABA; +R-ABA: treatment with rhizobia but no ABA; +R+ABA: treatment with both rhizobia and ABA

The nodule formation begins with the reciprocal recognition between legume plant and rhizobia, which leads to the formation of infection foci [1]. To test if ABA affects rhizobial infection, we analyzed the number of infection foci with or without ABA treatment. In the presence of 10 μM ABA, the number of infection foci per plant was dramatically reduced compared with that of plant without ABA treatment (Fig. 2a and b), suggesting that ABA inhibits the rhizobial infection in soybean.

The reciprocal recognition of legume plant and rhizobia activates downstream signaling events, leading to rhizobia-specific infection and nodule formation [1]. To test whether ABA affects rhizobial infection by interrupting nodulation signaling, we analyzed the transcript levels of the nodulation marker gene *GmENOD40–1* with or without ABA treatment. In the presence of rhizobia but without ABA treatment, *GmENOD40–1* expression was highly induced by rhizobia at 3 DAI, but with ABA treatment, the induction of *GmENOD40–1* was significantly reduced (Fig. 2c). The results indicate that ABA negatively regulates early nodulation through affecting the NF signaling transduction pathway.

### Phylogenetic and expression analysis of *ARBE* family genes in soybean

**Fig. 3:**
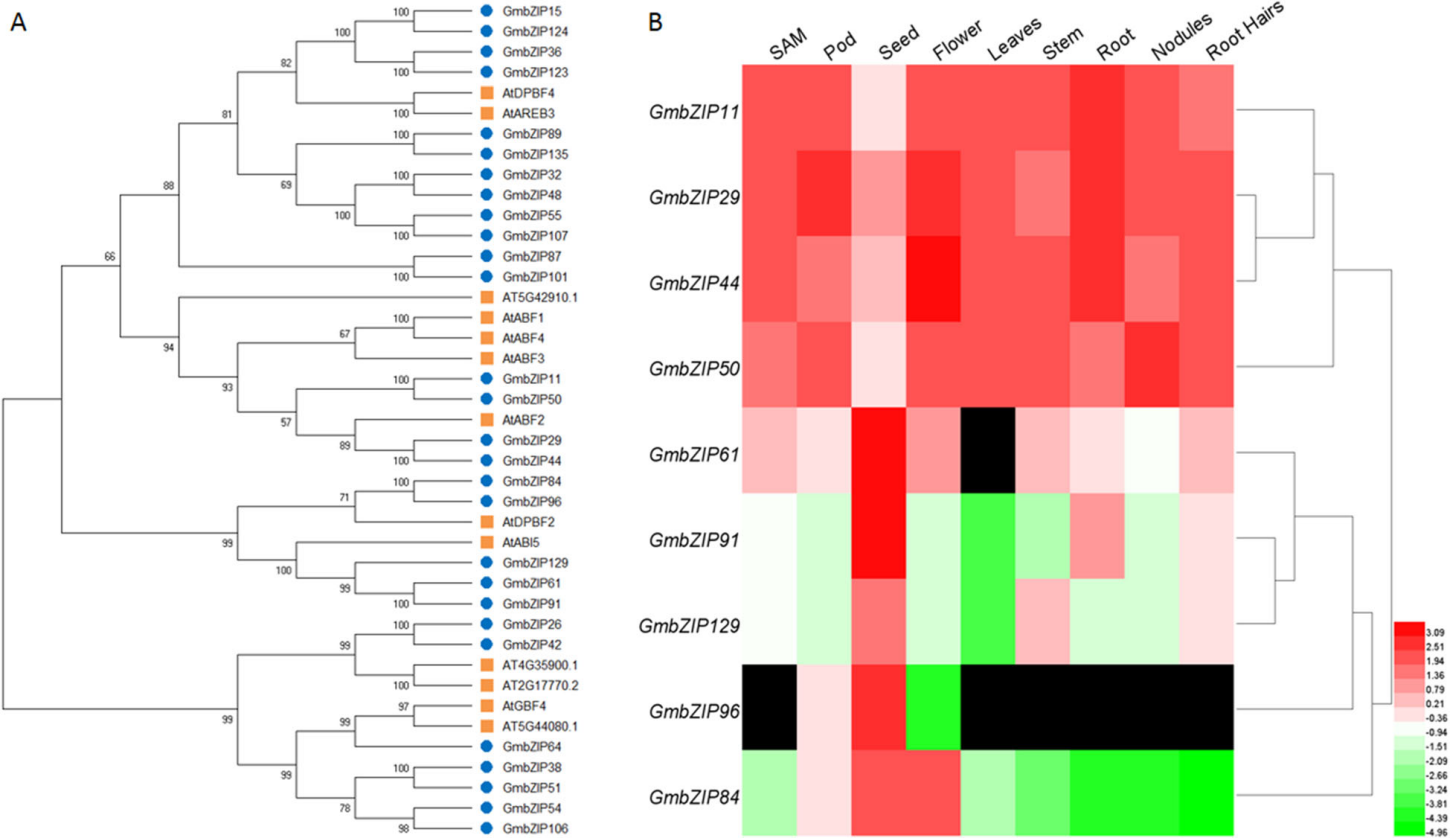
Phylogenetic and expression analysis of soybean AREB family genes. **a** Phylogenetic analysis of the group A bZIP family members in soybean and Arabidopsis. The amino acid sequences of the group A bZIP family member were used for the alignment, and the phylogenic neighbor-joining tree was constructed using MEGA5 phylogenetic analysis software. **b** Expression level analysis of AREB family gene members from public data (http://soybase.org)

AREB/ABF transcription factors are the central regulators of ABA responses in plants [43–45]. We wondered if the AREB transcription factors are involved in ABA-mediated suppression of soybean nodulation. AREB family genes encode the group A bZIP transcription factors and there are 28 group A members in soybean genome [49]. To search for the AREB transcriptional factors that involved in nodule organogenesis, we analyzed the evolutionary relationships between soybean and Arabidopsis. Similar to Arabidopsis [52], the 28 group A members in soybean can be classified in four distinct subgroups. Among them, GmbZIP11/ GmbZIP1 [50] (thereafter GmbZIP1), GmbZIP29, GmbZIP44 and GmbZIP50 are classified into the same clade with AtABF1-AtABF4 (Fig. 3a), which function in the ABA-mediated abiotic stresses response [43–45], while GmbZIP61, GmbZIP84, GmbZIP91, GmbZIP96 and GmbZIP129 are grouped in the same branch as Arabidopsis ABI5 (Fig. 3a), which regulates ABA-mediated seed maturation and seed germination [53]. The result suggests potential function of these GmZIP transcription factors in ABA response in soybean.

To find out the candidate *GmAREB* genes associated with nodulation, we analyzed the expression levels of *GmAREB* genes in different tissues and organs using RNA sequencing (RNA-Seq) data obtained from SoyBase [54] (http://soybase.org/). Among these *GmAREB* genes, *GmbZIP129* was nearly undetectable in all the tissues, four of them including *GmbZIP61*, *GmbZIP84*, *GmbZIP91*, *GmbZIP96* were predominantly expressed in seed, which are similar to their homologous gene *ABI5* in Arabidopsis [55]; and four genes including *GmbZIP1*, *GmbZIP29*, *GmbZIP44* and *GmbZIP50* were mainly expressed in flower and vegetative tissues including root hair and mature nodules (Fig. 3b). These data suggest that *GmbZIP1*, *GmbZIP29*, *GmbZIP44* and *GmbZIP50* may be involved in ABA-mediated nodule development in soybean.

### *GmbZIP1* is predominantly expressed during nodule formation and nodule development

**Fig. 4:**
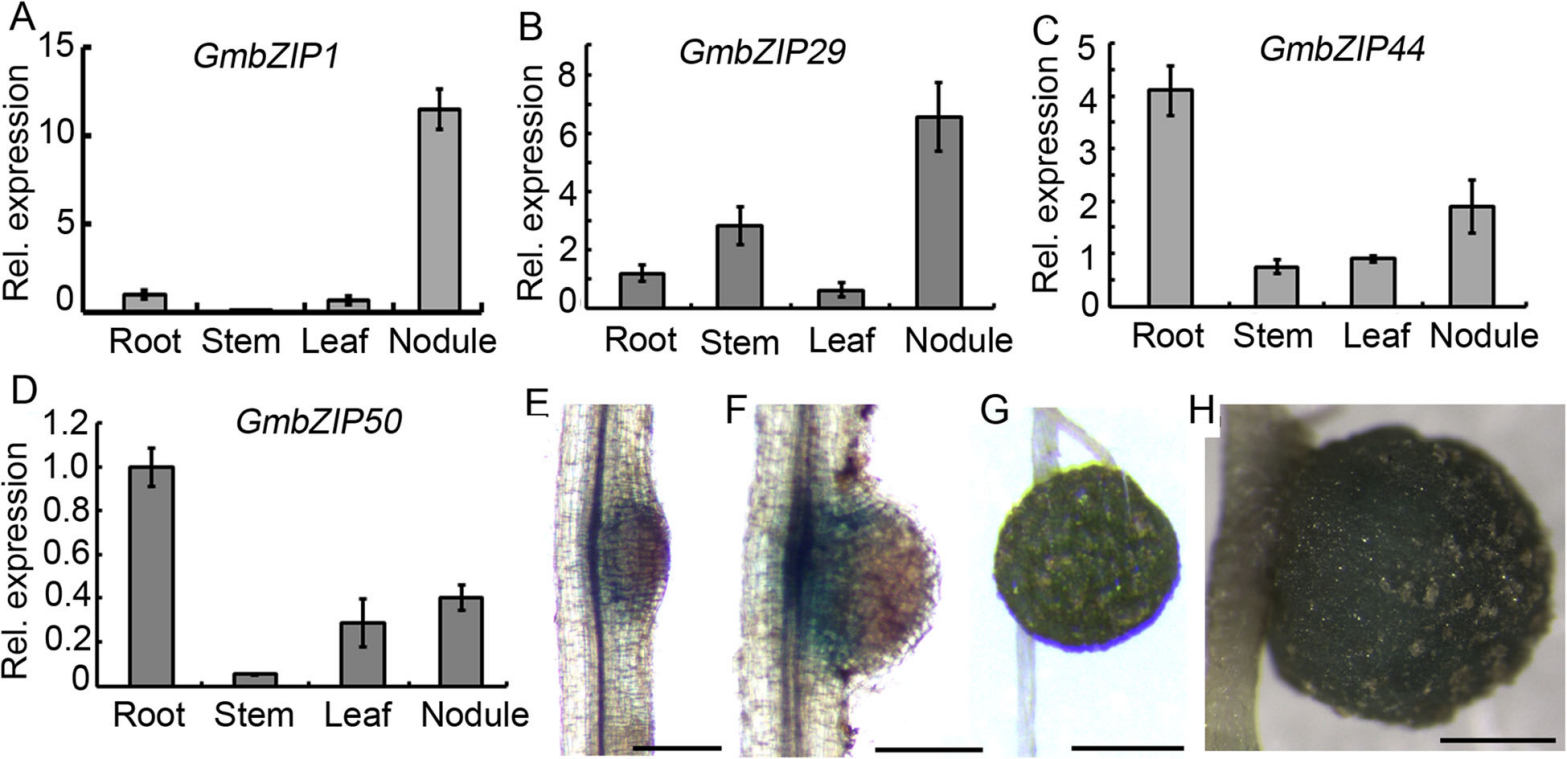
*GmbZIP1* is specifically expressed during soybean nodulation. **a** Expression pattern of *GmbZIP1* in roots, stem, leaves, and nodules harvested at 28. DAI. Seven-day-old seedlings were inoculated with *B. diazoefficiens* strain USDA110. **b** Expression pattern of *GmbZIP29* in roots, stem, leaves, and nodules harvested at 28 DAI. Seven-day-old seedlings were inoculated with *B. diazoefficiens* strain USDA110. **c** Expression pattern of *GmbZIP44* in roots, stem, leaves, and nodules harvested at 28 DAI. Seven-day-old seedlings were inoculated with *B. diazoefficiens* strain USDA110. **d** Expression pattern of *GmbZIP50* in roots, stem, leaves, and nodules harvested at 28 DAI. Seven-day-old seedlings were inoculated with *B. diazoefficiens* strain USDA110. *GmELF1b* was used as endogenous control, (**e**) *GmBZIP1pro:GUS* activity in nodule primordium at 3 DAI. Bar = 0.2 mm. **f** *GmBZIP1pro:GUS* activity in emerging nodule at 5 DAI, Bar = 0.2 mm. **g** GUS activity in young nodule at 14 DAI, Bar = 1 mm; **h** GUS staining of *GmBZIP1* in mature nodule at 28 DAI, Bar=5 mm

To identify the *GmAREB* gene(s) that specifically function(s) in soybean nodulation, we performed qPCR to validate the expression patterns of *GmbZIP1*, *GmbZIP29*, *GmbZIP44* and *GmbZIP50* in soybean. Indeed, these four genes were differentially expressed in the tested tissues/organs including mature nodule at 28 DAI (Fig. 4a-d), suggesting diverse roles of these four *GmAREB* genes during soybean growth and development. Among them, *GmbZIP44* and *GmbZIP50* were expressed at the highest levels in root but lower in leaf, stem and nodule (Fig. 4c and d), while *GmbZIP1* and *GmbZIP29* were expressed in all the tissues with the highest levels in nodule (Fig. 4a and b). Notably, *GmbZIP1* was predominantly expressed in nodule with very low or no expression in other organs, and its expression level in mature nodule was much higher than other *GmbZIP* genes (Fig. 4a). These results suggest that *GmbZIP1* may play a major and specific role in soybean nodule development.

To further examine the expression pattern of *GmbZIP1* during nodule formation and nodule development, we performed a histochemical analysis to test the promoter activity of *GmbZIP1* using the *GUS* (*β-glucuronidase*) gene as a reporter. To do this, the 3 kb fragment upstream of ATG of *GmbZIP1* was fused to the *gusA* gene and the resulting *GmbZIP1pro:GUS* construct was used for hairy root transformation and subsequent rhizobial nodulation. Histochemical analysis of *GmbZIP1pro:GUS* transgenic hairy roots was conducted at the specified stages of nodulation. High GUS activity was observed in nodule primordium and GUS activity was strongest in the cells at the base of nodule primordium (Fig. 4e). A high level of GUS activity was mainly observed at the base of emerging nodule (Fig. 4f), and GUS activity remained high in young nodule and mature nodules (Fig. 4g and h). These observations indicate that *GmbZIP1* expression is activated during nodule formation and nodule development.

### *GmbZIP1* is responsive to ABA and Positively regulates plant response to ABA

**Fig. 5:**
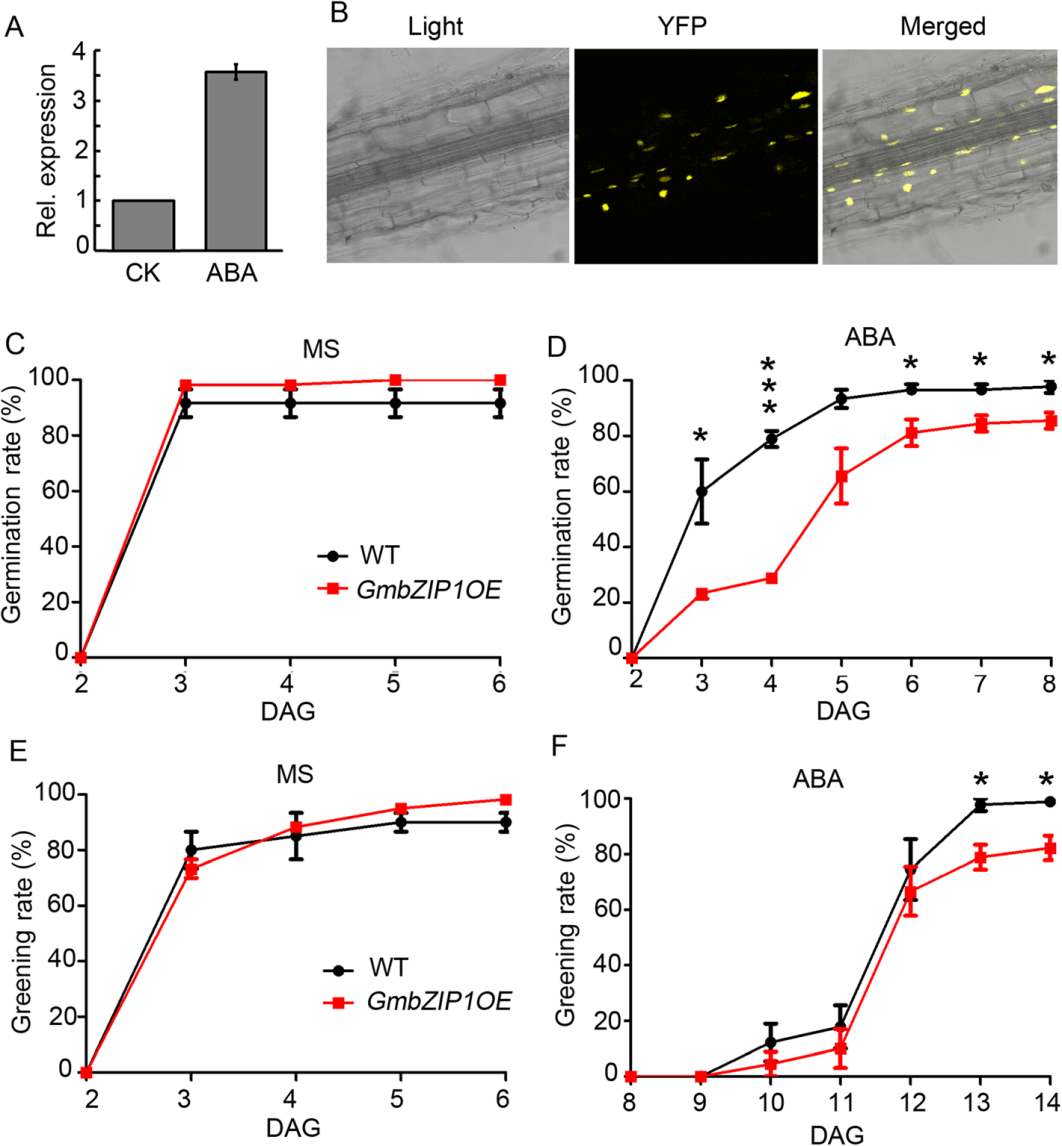
*GmbZIP1* is responsive to ABA. **a** qRT-PCR analysis of expression of *GmbZIP1* in the response to ABA. Five-day-old seedlings of soybean were treated with 20 μM ABA for three hours. *GmELF1b* was used as an endogenous control for gene expression. The expression levels are shown as the means ± SDs from three replicates. **b** The subcellular localization analysis of GmbZIP1. GmbZIP1 was fused to YFP and expressed in Arabidopsis. **c** Germination rates of wild type and *GmbZIP1* overexpression line on MS. **d** Germination rates of wild type and *GmbZIP1* overexpression line on MS supplemented with 0.5 μM ABA. * *P*< 0.05, *** *P*< 0.001. **e** Greening rates of wild type and *GmbZIP1* overexpression line on MS. **f** Greening rates of wild type and *GmbZIP1* overexpression line on MS supplemented with 0.5 μM ABA. * P< 0.05

It has been previously shown that *GmbZIP1* was induced by ABA in *G. max* L. cv. Tiefeng 8 and *GmbZIP1* overexpression in Arabidopsis resulted in increased ABA sensitivity of plants [50]. To see if *GmbZIP1* in Williams 82 is also ABA responsive, we analyzed the promoter of *GmbZIP1* from Williams 82 and found one ABRE *cis* elements in the 1507 bp (CACGT) upstream of ATG (Supplemental Fig. 1), suggesting that *GmbZIP1* expression may be regulated by ABA. Further qPCR assay results confirmed that *GmbZIP1* expression was indeed induced by ABA in 5 day-old soybean seedlings (Fig. 5a).

To investigate whether GmbZIP1 in Williams 82 has the conserved function in plant ABA response, we compared the coding sequences of *GmZIP1* genes between Tiefeng 8 and Williams 82, and found only six nucleotide difference between Tiefeng 8 and Williams 82 *GmbZIP1s*, which causes only four amino acid difference outside of the conserved domains in the corresponding proteins (Supplemental Fig. 2). To see whether the function of the GmbZIP1 from Williams 82 in ABA response remains unchanged, we also ectopically overexpressed *GmbZIP1-GFP* under control of the CaMV 35S promoter in Arabidopsis. As shown in Fig. 5b, high intensity of GFP signal was observed in nucleus of GmbZIP1-GFP transgenic root cells, suggesting that GmbZIP1 was successfully expressed in Arabidopsis (GmbZIP1OE) and GmbZIP1 is a nuclear protein. We then tested the ABA sensitivity of the Arabidopsis plants overexpressing *GmbZIP1*. In the absence of ABA, the rates of seed germination and cotyledon greening of *GmbZIP1OE* plant were similar to that of the wild type (Fig. 5c and e). By contrast, in the presence of ABA, both seed germination and cotyledon greening rates of *GmbZIP1OE* plant were significantly lower than that of wild type (Fig. 5d and f), suggesting that ectopic expression of *GmbZIP1* resulted in increased ABA sensitivity of Arabidopsis plants. ABA is a key regulator of plant drought tolerance [56]. To see whether GmbZIP1 affects plant response to drought stress, we also evaluated drought tolerance of *GmbZIP1OE* plants after withdrawing water and rehydration. Survival rate of *GmbZIP1OE* plants under drought conditions was much higher than that of wild type (Supplemental Fig. 3), which is consistent with the previous result about a role of GmbZIP1 in drought tolerance [50]. Taken together, these results demonstrate that *GmbZIP1* in Williams 82 is a positive regulator of plants in response to ABA response.

### *GmbZIP1* negatively regulates Rhizobial infection and nodule number

**Fig. 6:**
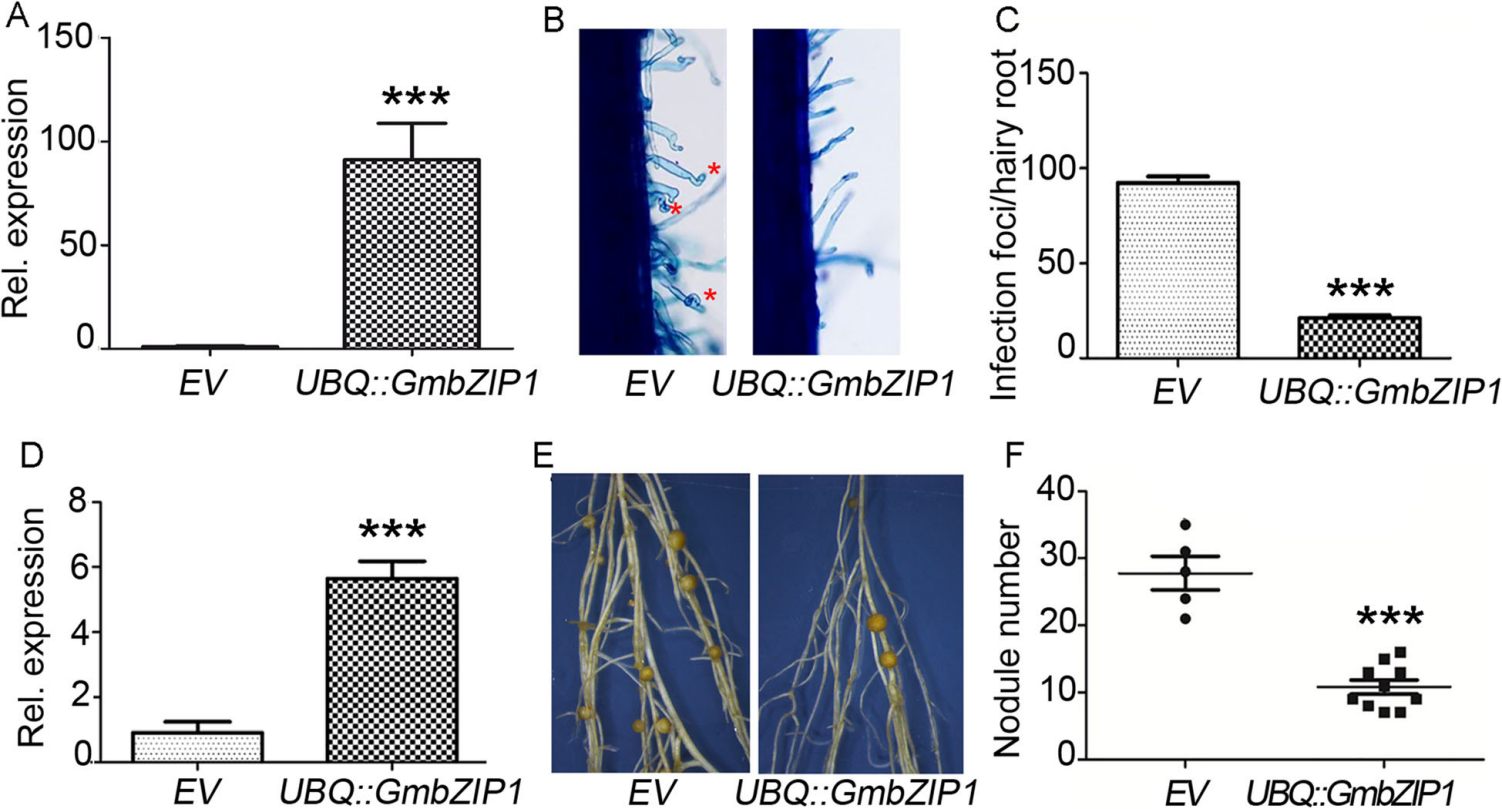
*GmbZIP1* overexpression inhibits soybean nodulation. **a** qRT-PCR analysis of *GmbZIP1* expression in transgenic empty or *UBQ*::*GmbZIP1* roots at 7 DAI. Values are mean ± SD, *p*< 0.001. **b** Infection foci of individual hairy roots expressing the empty vector and *UBQ*::*GmbZIP1*. At 7 DAI, 2-cm root segments of hairy roots overexpressing *GmbZIP1* below the root-hypocotyl junction were cut and stained with 1% (w/v) methylene blue. Considerably deformed root hairs were counted. The asterisk marks the curled root hair; **c** Quantitative analysis of the infection foci number per hairy root expressing empty vector or *UBQ*::*GmbZIP1* at 7 DAI. Values are mean ± SD, p< 0.001. **d** qRT-PCR analysis of *GmbZIP1* expression in transgenic empty vector or *UBQ*::*GmbZIP1* roots at 14 DAI. Values are mean ± SD, p< 0.001. **e**, **f** Overexpression of *GmbZIP1* decreased the number of nodulation. The nodule number was counted at 14 DAI of empty vector and *UBQ*::*GmbZIP1*

The next immediate question is whether *GmbZIP1* functions in rhizobial infection and nodule organogenesis in soybean. To do this, we generated transgenic composite plants overexpressing *GmbZIP1* under the control of the ubiquitin promoter (*UBQ::GmbZIP1*) (Fig. 6a), and examined the influence of *GmbZIP1* overexpression on root hair deformation and nodule number at early and late stages of nodulation. As shown in the result (Fig. 6b and c), the number of the deformed root hairs in *UBQ::GmbZIP1* hairy roots was enormously decreased compared with that in empty vector control at 7 DAI, suggesting a crucial role of *GmbZIP1* in early stage of rhizobial infection. We then scored the nodule number at 14 DAI, and found that the transgenic hairy roots overexpressing *GmbZIP1* produced fewer nodules than the empty vector control (Fig. 6d-f, Supplemental Fig. 4). These data reveal a negative role of *GmbZIP1* in rhizobial infection and nodule development.

### GmbZIP1 directly binds and represses expression of *GmENOD40–1*

**Fig. 7:**
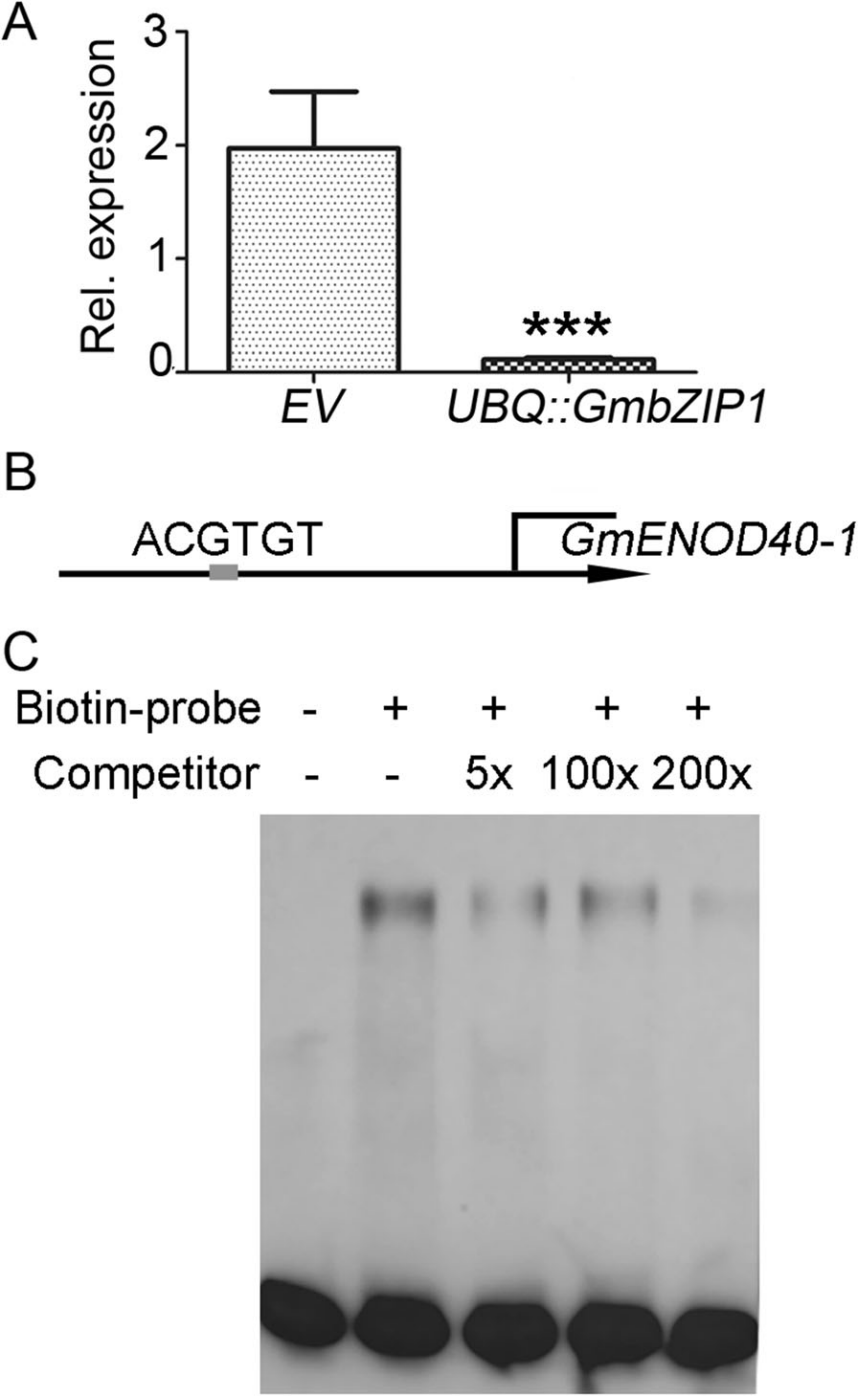
GmbZIP1 binds and represses *GmENOD40*–*1* expression. **a** qRT-PCR analysis of *GmENOD40–1* expression in transgenic empty or *UBQ*::*GmbZIP1* overexpression roots at 14 DAI. Values are mean ± SD, p< 0.001. **b** Schematic representation of *GmENOD40–1* locus. The *GmENOD40–1* promoter was shown as a line and the ACGT motif (ACGTGT) was shown as grey box. **c** EMSA for DNA binding domain of GmbZIP1 fused with a His tag to oligo-DNAs containing ACGTGT. Biotin-labeled probe was incubated with GmbZIP1-His. Competition for binding was performed with different concentration of unbiotin-labeled probe. The experiments were repeated for three times, and the representative results were shown

To see whether GmbZIP1 inhibits rhizobial infection and nodule development through the nodulation signaling pathway, we analyzed the effect of *GmbZIP1* overexpression on the well-known nodulation marker gene *GmENOD40–1* using qPCR [57]. Intriguingly, the expression level of *GmENOD40–1* in the *UBQ::GmbZIP1* transgenic roots was substantially reduced compared to the vector control at 14 DAI (Fig. 7a). This result indicates that GmbZIP1 modulates soybean nodulation through the nodulation signaling pathway.

GmbZIP1 is a putative transcription factor and we speculated that GmbZIP1 may directly bind to the promoter of *GmENOD40–1* and repress its expression or downregulates *GmENOD40–1* by targeting the upstream positive regulators in the nodulation pathway. To this end, we first analyzed *cis* elements in the 3 kb *GmENOD40–1* promoter. Interestingly, there was one AREB *cis* element in the promoter of *GmENOD40–1* (911 bp upstream of ATG) (Fig. 7b), suggesting a possible interaction between GmbZIP1 protein and the *GmENOD40–1* promoter. To prove the binding of GmbZIP1 to the *GmENOD40–1* promoter, we performed a DNA electrophoretic mobility shift assay (EMSA). For this, we expressed and purified the DNA binding domain of GmbZIP1 fused with a His tag in *Escherichia coli*. The result showed that the GmbZIP1-His fusion protein was able to bind to the DNA probes containing the AREB cis-element at the regions of the *GmENOD40–1* promoter (Fig. 7c, Supplemental Fig. 5). In a competition assay, the binding of GmbZIP1-His to the labeled DNA probe was reduced significantly by an excess amount of biotin-unlabeled DNA probe (Fig. 7c, Supplemental Fig. 5), suggesting that the interaction between GmbZIP1 and the *GmENOD40–1* promoter was specific. Collectively, these data suggest that GmBZIP1 regulates ABA-mediated nodulation inhibition by directly targeting *GmENOD40–1* and repressing its transcription.

## Discussion

Symbiotic nodulation is a complex trait that is precisely regulated by endogenous cues and environmental conditions. Phytohormones play an integral role in overall nodulation. Auxin and cytokinin positively regulate nodulation, while others negatively regulate nodulation. Therefore, dynamic hormonal balance is crucial to optimal and successful interaction between rhizobia and plants and nodule formation [36]. It has been shown that ABA application inhibits both indeterminate and determinate nodules in legumes [33, 35, 46]. However, how ABA regulates nodulation, in particular how ABA signaling interplays with nodulation signaling remains elusive. In this study, we showed that nodulation is the most sensitive process during plant growth and development; we identified GmbZIP1 as positive regulator of ABA response but negative regulator in soybean nodulation; and most importantly, we revealed that GmbZIP1 inhibits nodulation through directly repressing *GmENOD40–1* expression.

ABA is a key regulator of plant growth and development [56]. In soybean, we found that exogenous ABA has diverse roles on shoot growth, lateral root development and nodulation. Low level of ABA stimulated shoot growth, while high level of ABA inhibited growth of shoot. In comparison, lateral roots were insensitive to ABA in lower ABA concentration, and high concentration of ABA promoted lateral root development. Interestingly, root nodule exhibited extremely sensitivity to ABA and ABA treatment reduced nodule number in a dose-dependent manner. Notably, nodulation inhibition mediated by ABA in soybean is specific, as the low concentration ABA (0.5 μM) was sufficient to inhibit nodulation but had no effect on shoot and root growth (Fig. 1e). Exogenous ABA can interrupt rhizobia-induced root hair deformation and nodule number in soybean, supporting the notion that during nodulation, ABA mainly exerts its negative regulatory role in early stage of nodulation including rhizobial infection and nodule formation as proposed before in *M. trunctula* and *L. japonicum* [33, 35]. It is conceivable that the hypersensitivity of early nodulation to ABA is a conserved mechanism for attenuating nodulation signaling to maintain optimal nodule number in legumes.

Nodulation signaling pathway is responsible for rhizibial infection and nodule formation. It has been proposed that ABA inhibits rhizobial infection and nodule initiation through suppressing upstream signaling nodes in epidermis and activation of nodule formation genes, such as *ENOD11* gene [35]. Consistent with this, our results showed that ABA exerts its inhibitory role through a similar mechanism in soybean. For example, the induction of *GmENOD40–1* in soybean nodule formation was significantly reduced by ABA treatment (Fig. 2d). ABA regulates various biological processes mainly through ABA signaling. The previous results have identified Sensitivity to ABA 1(STA-1) as a key ABA-mediated suppressor of the initiation of Nod factor signaling in Medicago [35], although the underlying molecular mechanism is not yet clear. However, the gene that mediates ABA-induced inhibition of nodule formation and its action remains completely unknown. Here, we provide several pieces of evidence to show that AREB transcription factor GmbZIP1 is a repressor of *GmENOD40–1* to suppress nodule initiation induced by ABA.

AREB family proteins are key transcription factors in the ABA signaling pathway that regulate plant responses to ABA by targeting many genes related to plant growth and stress tolerance [44, 45]. Firstly, we found that among the ABA-related AREB/ABF genes in soybean, *GmbZIP1* is specifically expressed in nodule primorida and nodule (Fig. 4). Secondly, we proved that *GmbZIP1* is a positive regulator of plant ABA response. *GmbZIP1* is induced by ABA and ectopic expression of *GmbZIP1* increased ABA sensitivity of plant during early development and drought tolerance in Arabidopsis (Fig. 5, Supplemental Fig. 3). Thirdly, we demonstrated that GmbZIP1 negatively regulates soybean nodulation as overexpression of *GmbZIP1* resulted in reduced number of root hair deformation and nodules (Fig. 6). Finally, we showed that *GmbZIP1* can directly bind to the AREB element in the *GmENOD40–1* promoter to repress its expression (Fig. 7). It is possible that rhizobia induces ABA to activate *GmbZIP1* expression in cortex cells, which in turn represses *GmENOD40–1* expression resulting in suppression of nodule formation. Since *GmbZIP1* is expressed in other stages of nodulation and *GmbZIP1* overexpression can also affect root hair deformation, we do not exclude the possibility that GmbZIP1 regulates ABA-mediated other processes during nodulation. Since *GmbZIP1* is responsive and mediates plant response to abiotic stress [50], it is also possible that *GmbZIP1* mediates nodulation under abiotic stress.

## Conclusion

In summary, our data have identified a key component GmbZIP1 in ABA signaling of soybean that plays a negative role in soybean nodulation. Intriguingly, our findings reveal a novel mechanism by which GmbZIP1 mediates crosstalk between the ABA and NF signal transduction pathways during nodule initiation. Considering high level of conservation in ABA-mediated nodulation inhibition and bZIP1 transcription factor in legumes, our findings provide novel insights into elucidation of ABA regulation of symbiotic nodulation.

## Methods

### Plant and rhizobia growth, hairy root transformation, and *B. diazoefficiens* inoculation

Soybean [*G. max* (L.) Merrill cv. Williams 82] was used to clone and analyze the function of *GmbZIP1*. The seeds were obtained from Lab of Prof. Tianfu Han (Chinese Academy of Agricultural Sciences). Soybean seedlings were cultured in a growth room at 25–26 °C and 16 h/8 h light/dark conditions. For soybean hairy root transformation, healthy and uniform soybean seeds were planted in soil for 3 days in growth room. Germinating seedlings were used for hairy root transformation with *Agrobacterium rhizogenes* strain K599 containing various constructs as described previously [58]. For nodulation phenotype assays, the putative transgenic plants were transplanted to pots containing vermiculite and grown for 1 week to allow rooting. They were then inoculated with 30 ml suspension of *B. diazoefficiens* USDA110 (OD600 = 0.08) as described previously [58]. The individual root and the nodules were collected for molecular characterization of the transgenic roots and for nodule number quantification at the indicated time points (days after infection, DAI). Shanshan Song and Shimin Xu undertook the formal identification of the plant material used in this study and the material used in this study didn’t deposited in publicly available herbarium.

### ABA treatment

For ABA treatments, the experiments were done according to the Jone et al. (Jones et al., 2013) with minor modifications. Briefly, the soybean was germinated on medium containing 7% agar for 6 days. The plants were then transferred on plants containing Jensen’s medium (CaHPO_4_ 1 g/L, MgSO_4_.7H_2_O 0.2 g/L, K_2_HPO_4_ 0.2 g/L, NaCl 0.2 g/L, FeCl_3_.6H_2_O 0.1 g/L, H_3_BO_3_ 1 mg/L, ZnSO_4_.7H_2_O 1 mg/L, CuSO_4_.5H_2_O 0.5 mg/L, MnCl_2_.4H_2_O 0.5 mg/L, NaMnO_4_.2H_2_O 1 mg/L, agar 11.5 mg/L) supplemented with different concentrations of ABA with roots on the plates and the shoots out of the plate. The plants were inoculated with suspension of *B. diazoefficiens* USDA110 (OD_600_=0.1). The plants were cultured in growth room and the plates were wrapped with silver paper to keep root in dark. 18 days later, the nodule number, shoot and root phenotype were analyzed.

### Curled root hair assays

In order to examine the early infection events, the root segments (4 cm) below the hypocotyl–root junctions were cut from the transformed hairy roots at 7 DAI. The root fragments were rinsed briefly in sterile PBS buffer and stained with 0.01% methylene blue for 15 min and then washed three times with deionized water as described previously [57]. The stained transgenic roots were observed with a Leica biological microscope. The root hairs with tight curls were counted and referred to as ‘Infection foci’.

### RNA extraction and quantitative PCR analysis

Total RNA was extracted from wild type or composite plants using Trizol reagent according to the manufacturer’s protocol (Tiangen Biotech Co., Ltd., Beijing, China). The total RNA samples were treated with DNase I (Invitrogen) to remove contaminating genomic DNA and first-strand cDNA was synthesized using a FastQuant RT Kit (Tiangen Biotech). Quantitative polymerase chain reactions (qPCR) were performed using SuperReal PreMix Plus (SYBR Green; Tiangen Biotech). *GmELF1B* was used as the internal reference gene [57].

### Vector construction

For the overexpression construct of *GmbZIP1*, the full length *GmbZIP1* was cloned and inserted into the vector *pUBI:pCAMBIA1301* using the restriction enzymes *Xba*I/*Bam*HI; For the *GmbZIP1 promoter: GUS* reporter construct, the putative promoter region (2.0 kb) of *GmbZIP1* was amplified from cv. Williams 82 genomic DNA and inserted into the vector pCAMBIA1391 using the restriction enzymes *EcoR*I/*Bam*HI.

### EMSA

Electrophoretic Mobility Shift Assays (EMSAs) were performed using the Light Shift Chemiluminescent EMSA Kit (Pierce) according to the manufacturer’s protocol and as described by Wang et al. [57]. For the *GmbZIP1*-*His* construct, the coding sequence of the DNA binding domain of GmbZIP1 (901–1320 bp) was amplified and inserted into pET22b using *Nde*I/*Hind*III. The probe-binding activity of the protein was analyzed using oligonucleotides labeled with biotin at the 5’end (Sangon, China). After incubation at room temperature for 30 min, the protein-probe mixture was separated on a 6% polyacrylamide gel and transferred to a Biodyne B nylon membrane (Pall). Migration of the biotin-labeled probes was detected using streptavidin-horseradish peroxidase conjugates that bind to biotin and the chemiluminescent substrate according to the manufacturer’s protocol.

### Statistical analysis

The data including gene expression and phenotypic analysis in this study were analyzed using GraphPad Prism 5 (GraphPad Software, Inc., La Jolla, CA). The averages and Standard Deviations (SDs) of the data were calculated, and one-way ANOVA Student’s *t* test were performed to generate *p* values.

## Supplementary Information

**Additional file 1:.** Supplementary information contains Supplementary Fig. S1-S3.

## Data Availability

All data generated or analysed during this study are included in this published article and its supplementary information files. The datasets used and/or analysed during the current study are available from the corresponding author on reasonable request.

## Abbreviations

ABA: Abscisic acid
ABF: ABRE binding factor
ABRE: ABA-responsive element
bZIP: Basic region/leucine zipper
CCaMK: Calmodulin-dependent Kinase
CK: Cytokinin
CNGC15: Nuclear-localized cyclic nucleotide-gated channels
DMI1: Does Not Make Infections 1
ENOD11: Early Nodulin 11
ERN1: Ethylene Response Factor Required for Nodulation1
JA: Jasmonate
NF: Nod factor
NFR: NF receptor
NIN: Nodule Inception
PP2C: Group A protein type 2C phosphatases
RIP: Rhizobium-induced Peroxidase
SA: Salicylic acid
SnRK2s: Sucrose nonfermenting-1-related protein kinase class 2.
