# Supplementary material for: GmbZIP1 negatively regulates ABA-induced inhibition of nodulation by targeting GmENOD40–1 in soybean
Source: BMC Plant Biol. 2021 Jan 9;21:35. doi: 10.1186/s12870-020-02810-9 (PMC7796624; doi:10.1186/s12870-020-02810-9)
Supplement: Supplementary file 1 — Additional file 1:. Supplementary information contains Supplementary Fig. S1-S3. [file 12870_2020_2810_MOESM1_ESM.docx]

**Supplemental Figures**

**
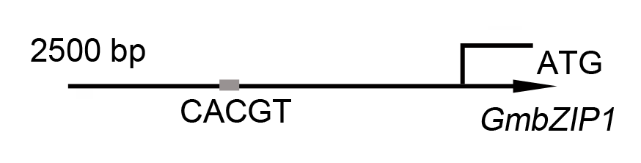
**

**Supplemental Figure 1.** **Schematic representation of *GmbZIP1* locus.**

The *GmbZIP1* promoter was shown as a line and the ACGT motif (CACGT) was shown as grey box.


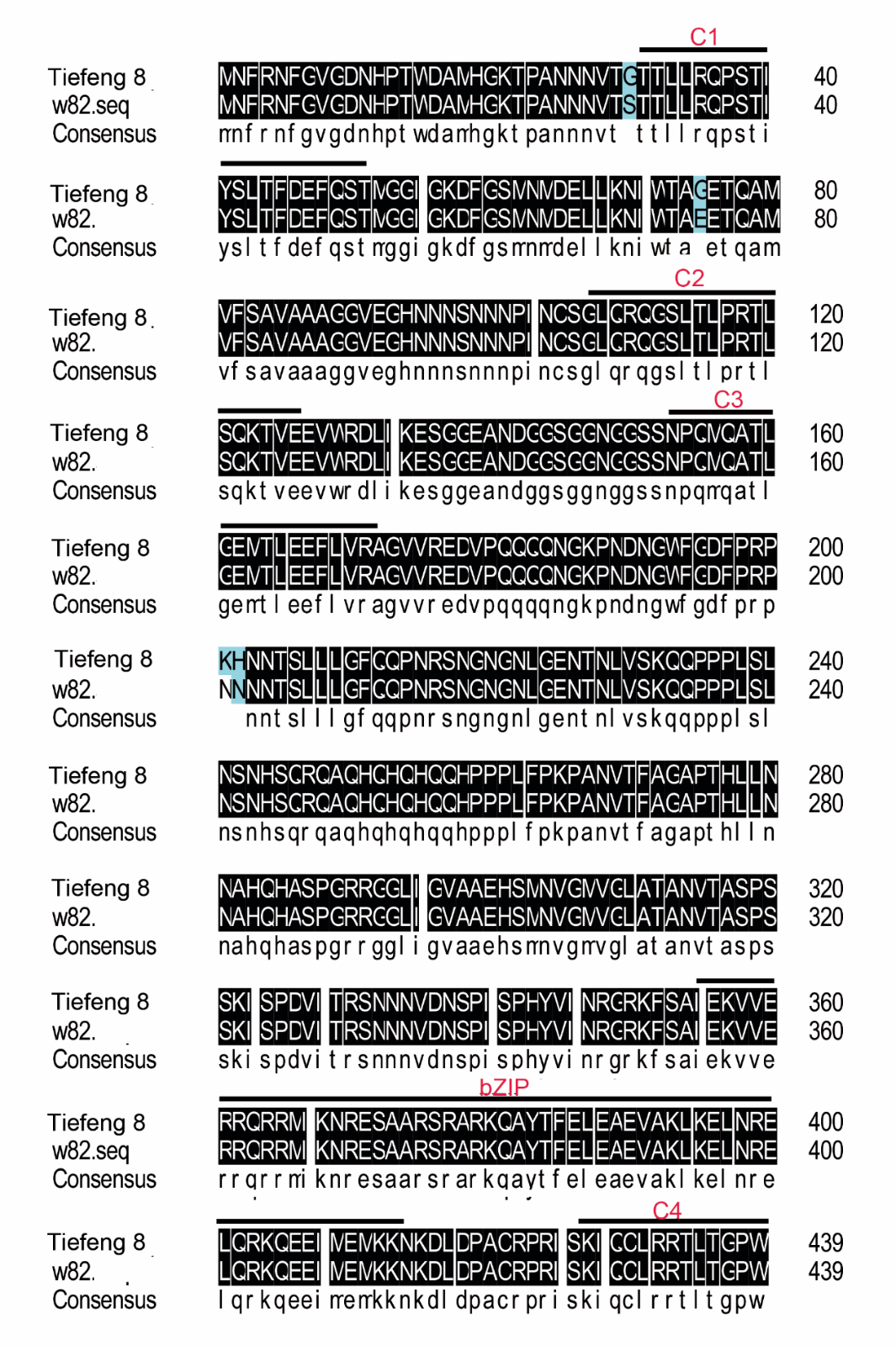


**Supplemental Figure 2.** Alignment of amino acid of GmbZIP1 from Williams 82 and Tiefeng 8. The difference amino acids were noted and the conserve domains (C1-C4, bZIP) were marked.


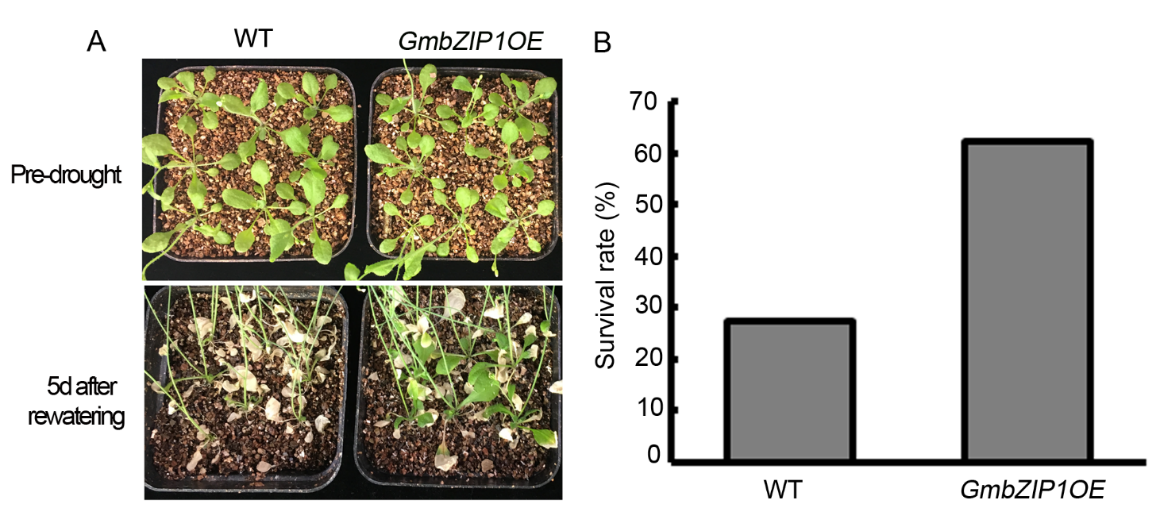


**Supplemental Figure 3.** *GmbZIP1* increases tolerant to dehydration stress.

(A) Drought tolerance assay of the wild-type (WT) and *GmbZIP1OE.* Water was withheld from 21-day-old plants. After 21 days of drought treatment, the plants were rewatered, and the plants were photographed 5 days after re-watering. The data represent the survival rate of the plants.

(B) Comparison of survival rate of wild type and *GmbZIP1OE* 5 d after rewatering.


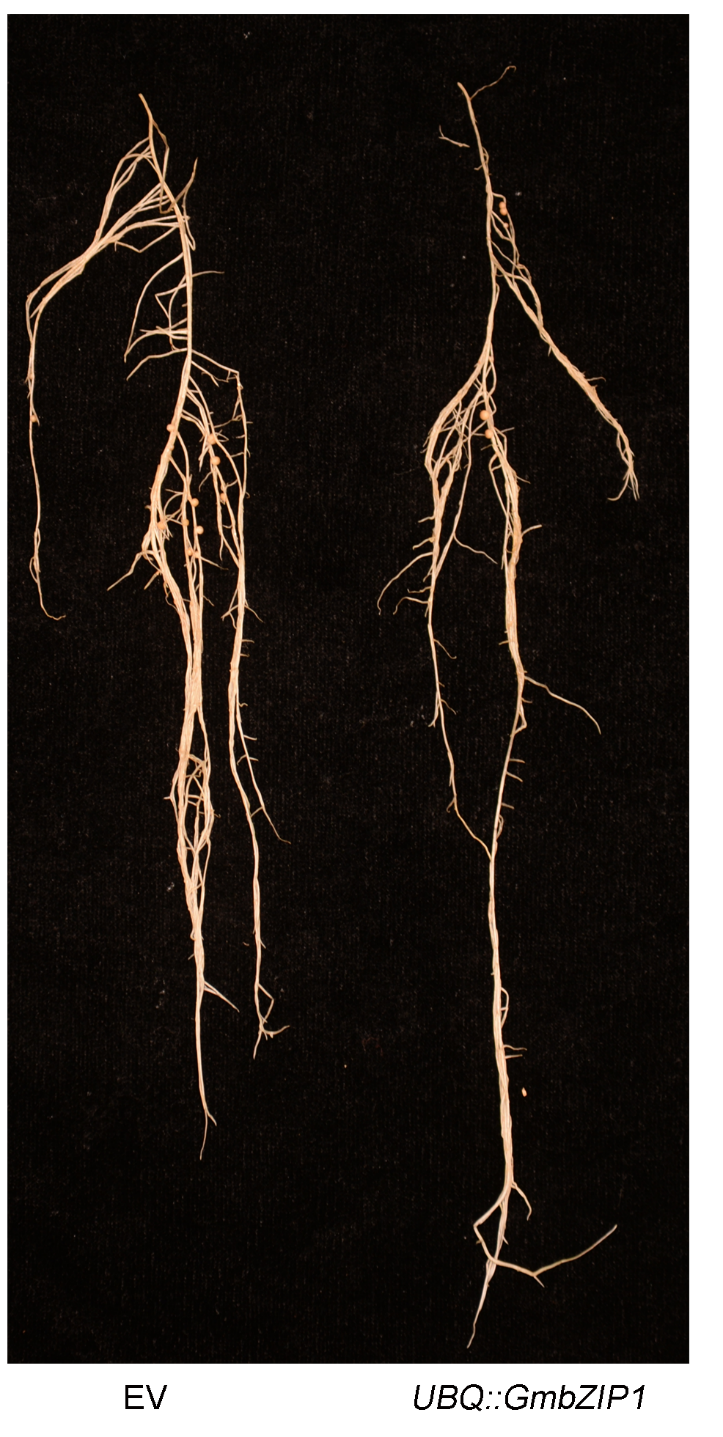


**Supplemental Figure 4.** Overexpression of *GmbZIP1* decreased the number of nodulation.

The nodule number was counted at 14 DAI of empty vector and *UBQ*::*GmbZIP1*.


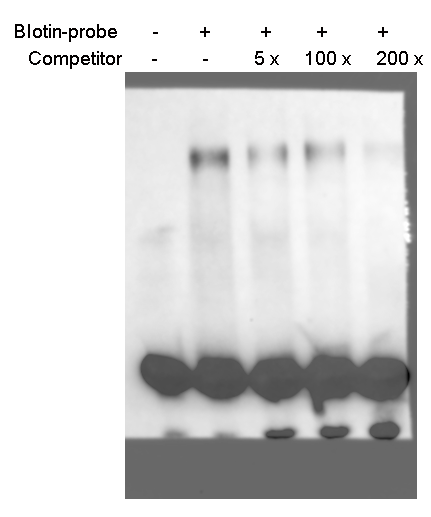


**Supplemental Figure 5.** GmbZIP1 binding to *GmENOD40-1* promoter.

Biotin-labeled probe was incubated with DNA binding domain of GmbZIP1 fused with a His tag. Competition for binding was performed with different concentration of unbiotin-labeled probe.
